# A Pre-Interventional Scale to Predict *in situ* Atherosclerotic Thrombosis in Acute Vertebrobasilar Artery Occlusion Patients

**DOI:** 10.3389/fneur.2021.648081

**Published:** 2021-04-07

**Authors:** Mingming Zha, Min Wu, Xianjun Huang, Xiaohao Zhang, Kangmo Huang, Qingwen Yang, Haodi Cai, Yachen Ji, Qiushi Lv, Dong Yang, Qiliang Dai, Rui Liu, Xinfeng Liu

**Affiliations:** ^1^Department of Neurology, Jinling Hospital, Medical School of Southeast University, Nanjing, China; ^2^Department of Neurology, Jinling Hospital, The First School of Clinical Medicine, Southern Medical University, Nanjing, China; ^3^Department of Neurology, The First Affiliated Hospital of Wannan Medical College, Wuhu, China; ^4^Department of Neurology, Jinling Hospital, Affiliated Medical School of Nanjing University, Nanjing, China

**Keywords:** endovascular treatment, vertebrobasilar artery occlusion, *in situ* atherosclerotic thrombosis, acute ischemic stroke, predictive model

## Abstract

**Background and Purpose:** Determining the occlusion mechanism before endovascular treatment (EVT) is of great significance for acute large vessel occlusion patients. We aimed to develop and validate a simple pre-EVT scale with readily available variables for predicting *in situ* atherosclerotic thrombosis (ISAT) in acute vertebrobasilar artery occlusion (VBAO) patients.

**Materials and Methods:** Consecutive patients were retrieved from Nanjing Stroke Registry Program between January 2014 and December 2019 as a derivation cohort. Anonymous data of consecutive patients between January 2014 and December 2019 were collected from another comprehensive stroke center as an external validation cohort. Demographics, medical histories, and clinical characteristics were collected. ISAT was defined according to the following criteria: (a) detection of moderate to severe (≥50%) stenosis or stenosis with significant distal flow impairment at the occluded segment when successful reperfusion was achieved; (b) transient visualization of eccentric plaque contour or a recurrent re-occlusion tendency when reperfusion was unsuccessful. Logistic regression was taken to develop a predictive scale. The performance of the scale was assessed by area under the receiver operating characteristic curve (AUC) and Hosmer–Lemeshow test.

**Results:** ISAT was observed in 41 of 95 (43.2%) patients included in the derivation cohort. The ISAT predictive scale consisted of three pre-interventional predictors, including the history of hypertension, atrial fibrillation rhythm, and baseline serum glucose level ≥7.55 mmol/L. The model depicted acceptable calibration (Hosmer–Lemeshow test, *P* = 0.554) and good discrimination (AUC, 0.853; 95% confidence interval, 0.775–0.930). The optimal cutoff value of the ISAT scale was 1 point with 95.1% sensitivity, 64.8% specificity, and 77.9% accuracy. In the validation cohort, the discrimination ability was still promising with an AUC value of 0.800 (0.682–0.918).

**Conclusion:** The three-item scale comprised of the history of hypertension, atrial fibrillation rhythm, and dichotomous serum glucose level had a promising predictive value for ISAT before EVT in acute VBAO patients.

## Introduction

Acute vertebrobasilar artery occlusion (VBAO) is one of the most devastating types of acute ischemic stroke with a high disability and mortality rate (1). The best treatment choice for VBAO is still under debate (2), but evidence on the effectiveness of endovascular treatment (EVT) in treating VBAO is accumulating (3–5).

Considering the heterogenicity in occlusion mechanisms, VBAO patients can be categorized into different subtypes (6, 7). Different occlusion mechanisms exert influences on device selections, reperfusion procedures, and clinical prognosis (8–10). One of the most frequent causes of VBAO is atherosclerotic occlusion resulting from local thrombosis due to severe stenosis (1). Previous studies indicate that intracranial atherosclerosis-related occlusion has a higher intraprocedural re-occlusion rate, need for rescue therapies, and longer puncture-to-reperfusion time (11). Figuring out the exact occlusion mechanism of VBAO is beneficial for EVT procedures.

Analyses and predictions of occlusion types have been investigated in previous studies (12, 13). However, these studies usually rely heavily on digital subtraction angiography (DSA) characteristics, which seems lagging in predicting models. Simple predictive scales with readily available parameters before EVT are needed.

Thus, we performed a retrospective analysis on consecutive patients from a prospectively enrolled stroke database to develop a pre-EVT predictive scale for ISAT in VBAO patients and tested its performance in a cohort enrolled from another comprehensive stroke center.

## Methods

### Patient Selection

De-identified data collected from the Nanjing Stroke Registry Program were taken as the derivation cohort. Nanjing Stroke Registry Program is a prospectively maintained database based on Jinling Hospital. Detailed introductions on this registry have been published previously (14). The validation cohort was established based on de-identified data from the Neurology Department of the Yijishan Hospital.

Between January 2014 and December 2019, angiographically proved acute VBAO patients who underwent EVT (e.g., intra-arterial thrombolysis/glycoprotein IIb/IIIa inhibitor, mechanical thrombectomy, angioplasty, or various combinations of these) were included. Patients who had non-occlusion, sub-occlusion, and estimated occlusion time to puncture >24 h were excluded. Patients were also excluded if EVT procedures were aborted owing to an inability to advance the guidewire, catheter, or other EVT devices to the occlusion site and without endovascular rescue therapies. Analyses on the Nanjing Stroke Registry Program were approved by the ethical committee of Jinling Hospital. Informed consent was waived due to its retrospective design.

### Data Collection

Demographic characteristics, medical histories, results of electrocardiogram examination on admission, imaging scale score, and lab results were collected and double-checked by two neurologists (MZ and MW). Severe stroke at the onset was defined when coma, quadriplegia, and locked-in syndrome were presenting symptoms (15). Prodrome was defined as stroke-associated symptoms before index events (coma, quadriplegia, and locked-in syndrome) of VBAO (16). Occlusion mechanisms were diagnosed according to established criteria (7), and ISAT was defined according to the following criteria: (a) detection of moderate to severe (≥50%) stenosis or stenosis with significant distal flow impairment at the occluded segment when successful reperfusion was achieved; (b) transient visualization of eccentric plaque contour or a recurrent re-occlusion tendency when reperfusion was unsuccessful. The embolism mechanism was diagnosed when there was no evidence of ISAT, including (a) complete recanalization without residual stenosis in occluded segments and (b) have established source of embolism with or without reperfusion (8).

Diagnoses of ISAT were independently finished by two experienced neurologists (MZ and MW) following the flowchart in the published reference (7), and disparities were solved by an experienced neuro-interventionalist (RL). Interobserver agreement for ISAT was assessed using Cohen's kappa coefficient (Cohen κ). Scores of the baseline National Institute of Health Stroke Scale (NIHSS) (17), the Glasgow Coma Scale (GCS) (18), and the modified Rankin Scale (mRS) (19) were collected. posterior circulation Acute Stroke Prognosis Early Computed Tomography Score (pc-ASPECTS) (20) was used to analyze brain ischemia before EVT.

### Statistical Analyses

Multiple imputations with chain equations were performed to account for missing values (≤10%). Patients were categorized into the ISAT group and the embolism group. Continuous data were expressed as mean (standard deviation, SD) if normally distributed or median (interquartile range, IQR) if not. Categorical data were expressed as number (percentage). Clinical parameters between the ISAT group and the embolism group were compared with *t*-test, Mann–Whitney U test, Chi-square test, and Fisher exact test as appropriate.

Candidate variables with *P*-value < 0.1 on univariate analysis were included in multivariable regression. Continuous variables were transformed into dichotomous variables to facilitate application before enrolling in the regression model, and the cutoff values were calculated with the receiver operating characteristic (ROC) curve by maximizing the Youden index (sensitivity + specificity − 1). Colinearity diagnosis was performed by using the variance inflation factor (VIF). Binary logistic regression (forward, likelihood ratio) was taken to generate the regression model. The discrimination and calibration of the model were assessed by the area under the ROC curve (AUC) and the Hosmer–Lemeshow test, respectively. β-coefficients obtained from the regression model were rounded to the closest integer and used to generate the scoring system of the ISAT scale (21). ROC curve analysis was used to calculate the optimal cutoff value of the ISAT scale.

Two-sided *P*-values < 0.05 were considered statistically significant. Analyses were performed using the SPSS software package, version 25 (IBM-Armonk, NY) and R statistical software, version 3.6.3 (22).

## Results

### Overview

**Figure 1:**
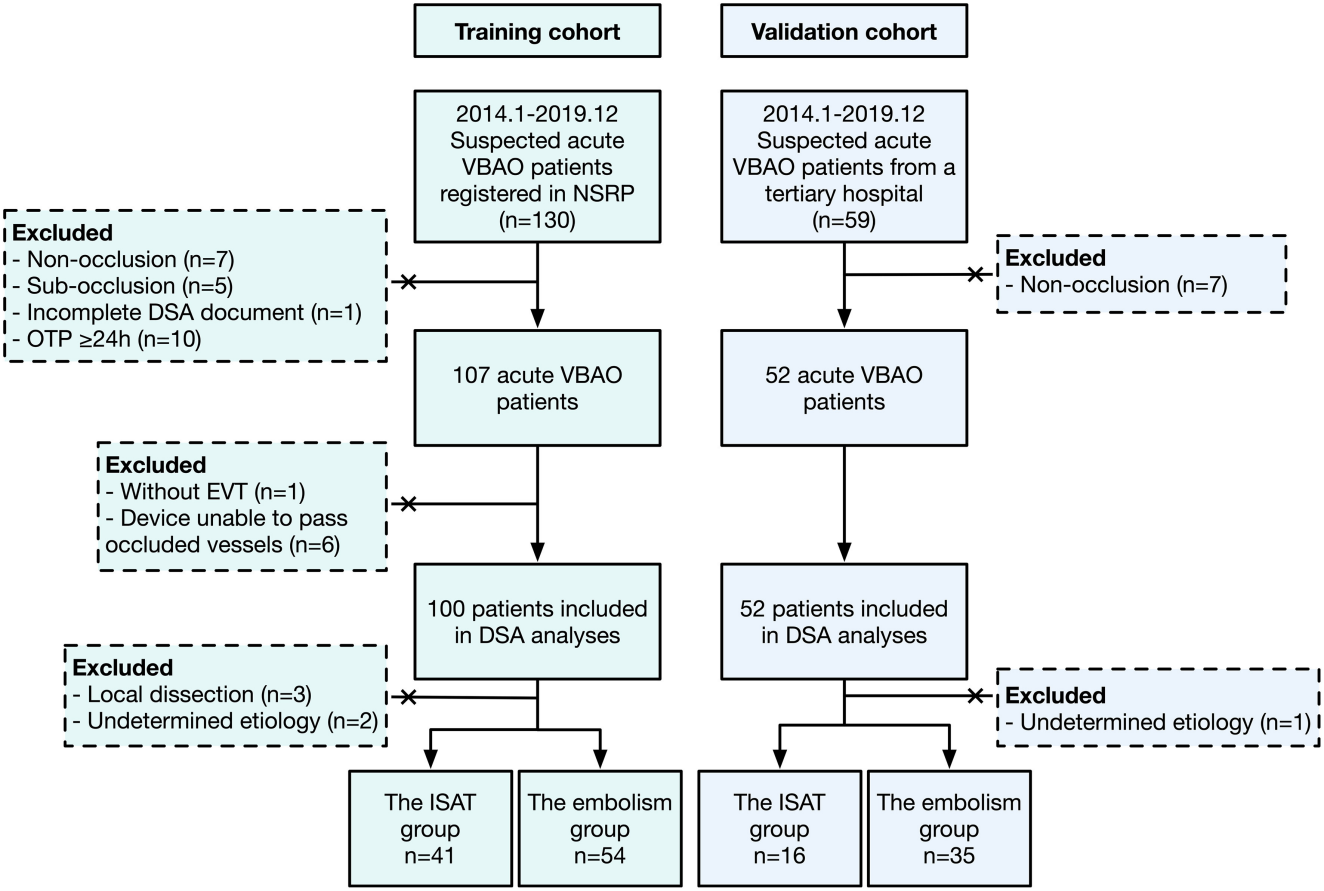
The flowchart of this study. VBAO, vertebrobasilar artery occlusion; NSRP, Nanjing Stroke Registry Program; DSA, digital subtraction angiography; OTP, onset to puncture; EVT, endovascular treatment; ISAT, *in situ* atherosclerotic thrombosis.

Between January 2014 and December 2019, 95 patients in the derivation cohort fulfilled the criteria and were enrolled in the final analyses (Figure 1). The mean age was 62.2 years old, and men accounted for 75.8% (72/95). Median (IQR) baseline NIHSS and GCS score were 26.0 (17.5, 29.5) and 6.0 (6.0, 11.0), respectively. The median mRS score on admission was 5.0 (4.0, 5.0). Ten (10.5%) patients had a history of coronary heart disease. Thirty-five (36.8%) patients had a smoking history, and the prevalence of hypertension history was 69.5%. Sixteen (16.8%) patients were diagnosed with atrial fibrillation (AF) previously, and electrocardiograms (ECGs) on admission indicated that 24 (25.3%) patients had AF rhythm. Twenty-seven (28.4%) patients had severe stroke at the onset. According to the appearances of DSA documents, 41 (43.2%) patients were categorized into the ISAT group and 54 (56.8%) into the embolism group. There was a good agreement on the presence of ISAT (Cohen κ: 0.782 in the derivation cohort and 0.783 in the validation cohort) between two independent neurologists.

### Predictors of *in situ* Atherosclerotic Thrombosis

Table 1 illustrates the comparisons of clinical parameters between the ISAT group and the embolism group. The average age in the ISAT group was significantly lower than that in the embolism group (59.5 vs. 64.3 years old, *P* = 0.039). The ISAT group had a lower percentage of AF history (2.4% vs. 27.8%, *P* = 0.001) and a higher proportion of hypertension history (85.4% vs. 57.4%, *P* = 0.003) when compared with the embolism group. Evaluations of stroke severity according to medical histories illustrated that fewer patients in the ISAT group had severe stroke at the onset (12.2 vs. 40.7%, *P* = 0.002). Median baseline NIHSS score of the patients in the ISAT group was statistically lower than that in the embolism group (25.0 vs. 27.0, *P* = 0.044), while the GCS score was comparable between the two groups (6.0 vs. 6.0, *P* = 0.113). Table 2 shows the comparisons of admission laboratory tests between the two groups. Median glucose level (7.7 vs. 6.8 mmol/L, *P* = 0.018), median blood urea nitrogen level (5.6 vs. 4.8 mmol/L, *P* = 0.047), and average total cholesterol level (4.92 vs. 4.39 mmol/L, *P* = 0.036) before EVT were significantly higher in the ISAT group.

**Table 1:** Demographics and clinical characteristics between the *in situ* atherosclerotic thrombosis group and the embolism group.

**Variable**	**The embolism group** **(*n* = 54)**	**The ISAT group** **(*n* = 41)**	***P-value***
Age, year, mean (SD)	64.3 (11.4)	59.5 (10.9)	0.039
Male, *n* (%)	37 (68.5)	35 (85.4)	0.058
SBP, mmHg, median [IQR]	145.0 [129.2, 158.0]	149.0 [133.0, 170.0]	0.245
DBP, mmHg, median [IQR]	80.0 [73.2, 89.8]	86.0 [78.0, 97.0]	0.021
**Medical history**			
Coronary heart disease, *n* (%)	7 (13.0)	3 (7.3)	0.507
AF, *n* (%)	15 (27.8)	1 (2.4)	0.001
Hypertension, *n* (%)	31 (57.4)	35 (85.4)	0.003
Hyperlipidemia, *n* (%)	1 (1.9)	4 (9.8)	0.162
Diabetes mellitus, *n* (%)	9 (16.7)	10 (24.4)	0.351
Acute ischemic stroke, *n* (%)	8 (14.8)	10 (24.4)	0.238
Intracranial hemorrhage, *n* (%)	2 (3.7)	1 (2.4)	1.000
Smoking, *n* (%)	19 (35.2)	15 (36.6)	0.888
Prodrome, *n* (%)	19 (35.2)	18 (43.9)	0.388
Severe stroke at the onset, *n* (%)	22 (40.7)	5 (12.2)	0.002
AF rhythm, *n* (%)	23 (42.6)	1 (2.4)	<0.001
Admission NIHSS score, median [IQR]	27.0 [18.2, 30.0]	25.0 [12.0, 27.0]	0.044
Admission GCS score, median [IQR]	6.0 [4.5, 8.8]	6.0 [6.0, 13.0]	0.113
Admission mRS score, median [IQR]	5.0 [4.0, 5.0]	5.0 [4.0, 5.0]	0.094
pc-ASPECTS, median [IQR]	8.0 [8.0, 9.0]	8.0 [7.0, 9.5]	0.639

*ISAT, in situ atherosclerotic thrombosis; SD, standard deviation; SBP, systolic blood pressure; IQR, interquartile range; DBP, diastolic blood pressure; AF, atrial fibrillation; NIHSS, National Institute of Health Stroke Scale; GCS, Glasgow Coma Scale; mRS, modified Rankin Scale; pc-ASPECTS, posterior circulation Acute Stroke Prognosis Early Computed Tomography Score*.

**Table 2:** Comparisons of laboratory tests between the *in situ* atherosclerotic thrombosis group and the embolism group.

**Variable**	**The embolism group (*n* = 54)**	**The ISAT group (*n* = 41)**	***P-value***
White blood cell, ^*^10^∧^9/L, median [IQR]	8.6 [7.1, 12.3]	10.2 [7.9, 12.0]	0.286
Neutrophil-lymphocyte ratio, median [IQR]	6.17 [3.96, 11.38]	5.98 [3.66, 8.86]	0.578
Platelet count, ^*^10^∧^9/L, mean (SD)	203.1 (69.3)	212.0 (66.4)	0.533
Glucose level, mmol/L, median [IQR]	6.8 [5.8, 8.0]	7.7 [6.3, 10.4]	0.018
Glycosylated hemoglobin, %, median [IQR]	6.1 [5.6, 6.6]	6.0 [5.6, 7.1]	0.850
C-reactive protein, mg/L, median [IQR]	4.0 [1.9, 10.5]	4.5 [1.4, 7.6]	0.693
ALT, U/L, median [IQR]	28.5 [21.0, 35.8]	25.0 [19.0, 39.0]	0.545
AST, U/L, median [IQR]	26.0 [21.2, 31.0]	22.0 [19.0, 29.0]	0.117
Blood urea nitrogen, mmol/L, median [IQR]	4.8 [4.1, 5.9]	5.6 [4.8, 6.4]	0.047
Creatinine, μmoI/L, median [IQR]	63.5 [55.0, 77.0]	70.0 [58.0, 78.0]	0.166
Total cholesterol, mmol/L, mean (SD)	4.39 (1.02)	4.92 (1.40)	0.036
Triglyceride, mmol/L, median [IQR]	1.16 [0.90, 1.48]	1.30 [1.00, 1.82]	0.130
Blood urine acid, μmoI/L, mean (SD)	333.1 (120.4)	312.9 (97.8)	0.382
Thrombin time, s, median [IQR]	17.6 [16.4, 19.4]	17.7 [16.1, 19.4]	0.952
Prothrombin Time, s, median [IQR]	11.9 [11.2, 12.6]	11.7 [11.3, 12.4]	0.676
APTT, s, median [IQR]	24.4 [22.8, 27.3]	24.3 [22.0, 28.6]	0.795
Fibrinogen, g/L, median [IQR]	2.96 [2.54, 3.46]	3.22 [2.74, 4.03]	0.129
D-dimer, mg/L, median [IQR]	1.05 [0.60, 2.98]	0.84 [0.36, 2.03]	0.293
International normalized ratio, median [IQR]	1.03 [0.97, 1.10]	1.02 [0.98, 1.06]	0.599

*ISAT, in situ atherosclerotic thrombosis; IQR, interquartile range; SD, standard deviation; ALT, alanine aminotransferase; AST, aspartate aminotransferase; APTT, activated partial thromboplastin time*.

### Formation and Assessment of the Predictive Scale

**Figure 2:**
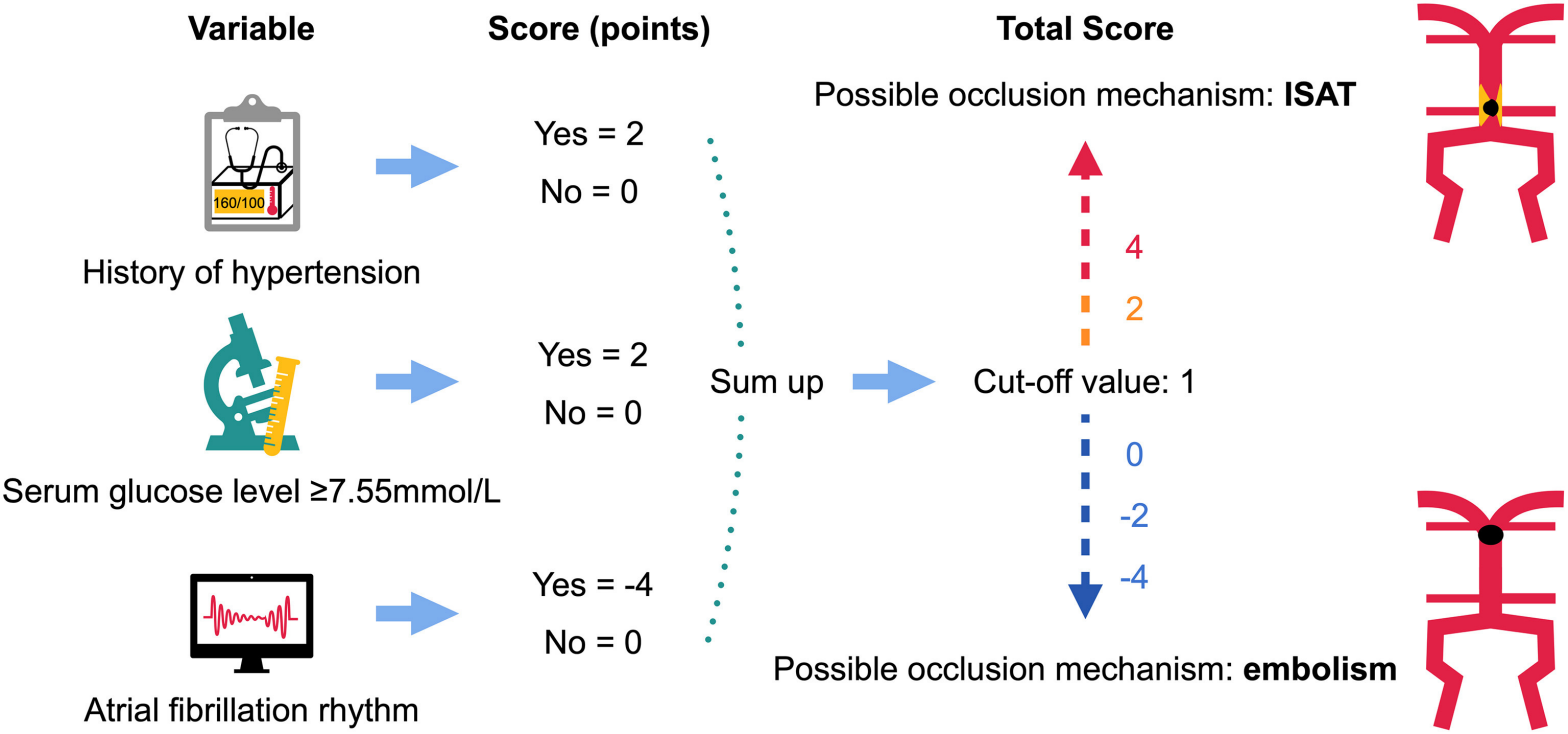
The illustration of the predictive scale on *in situ* atherosclerotic thrombosis. ISAT, *in situ* atherosclerotic thrombosis.

**Figure 3:**
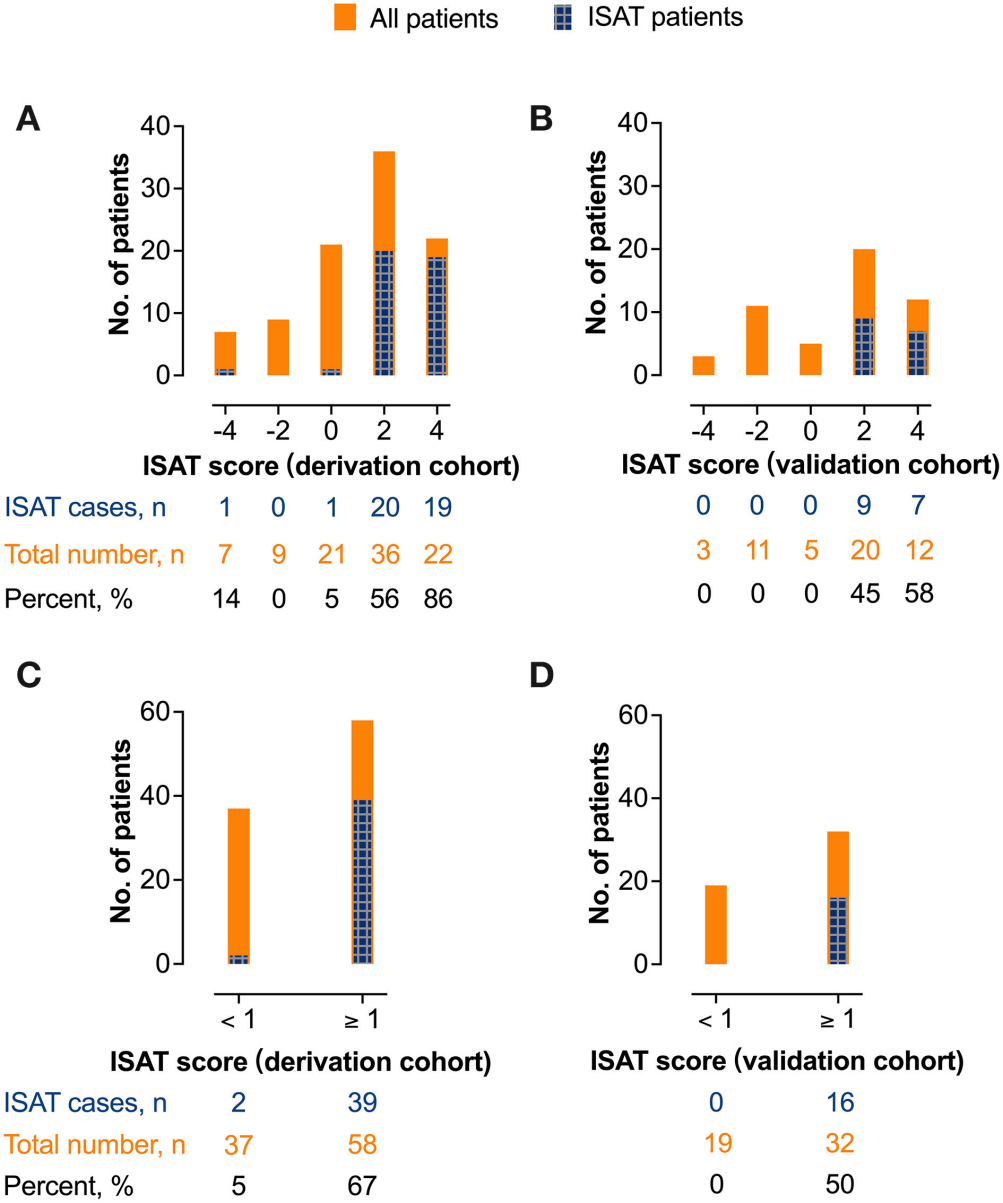
The distributions and percentages of *in situ* atherosclerotic thrombosis patients in different predictive scale risk categories. **(A)** The derivation cohort (consecutive). **(B)** the validation cohort (consecutive). **(C)** the derivation cohort (dichotomous). **(D)** the validation cohort (dichotomous). ISAT, *in situ* atherosclerotic thrombosis.

**Figure 4:**
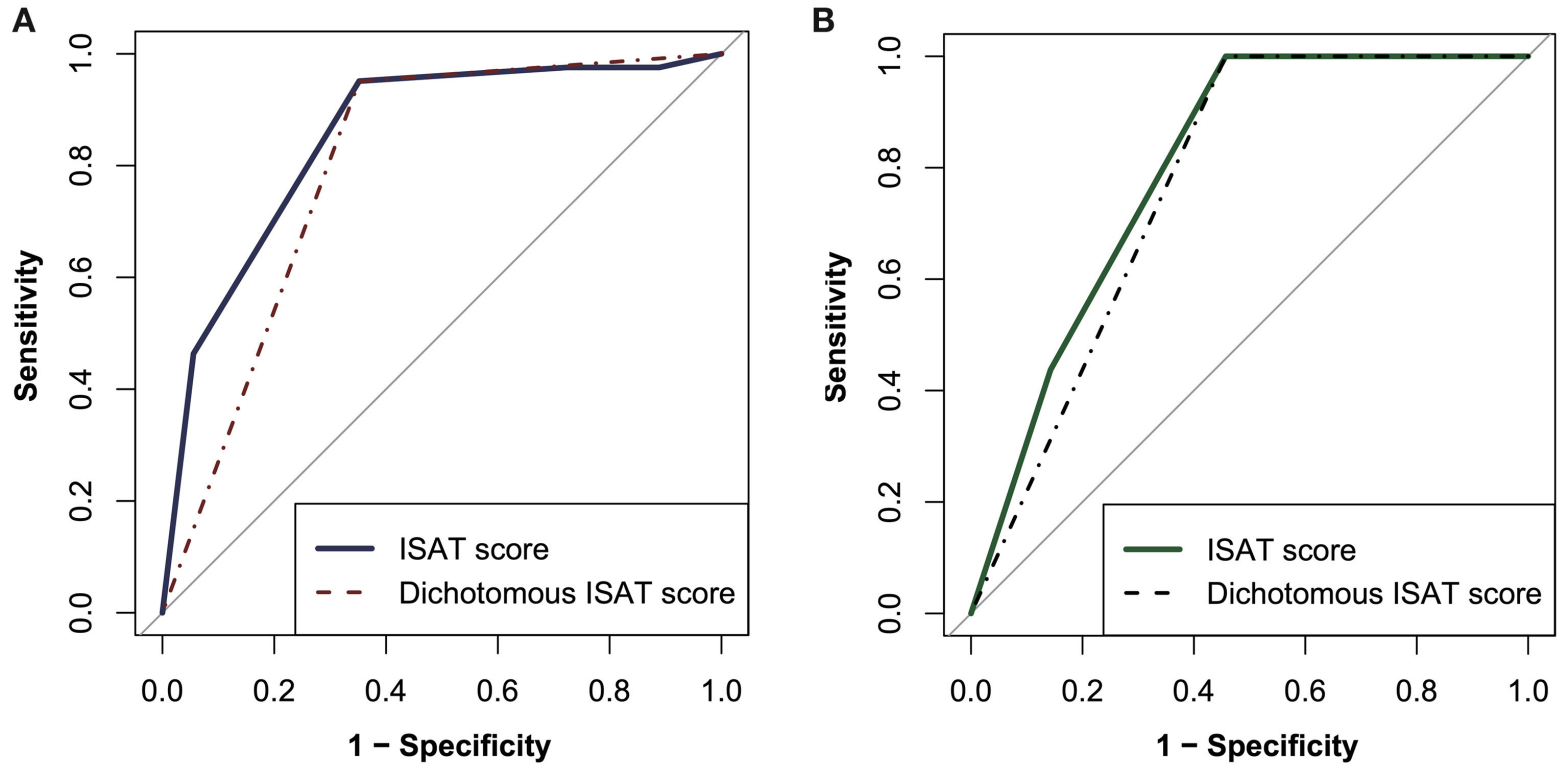
The receiver operating characteristic curves of the consecutive and dichotomous predictive scale. **(A)** The derivation cohort. **(B)** the validation cohort. ISAT, *in situ* atherosclerotic thrombosis.

After excluding colinearity and enrolling all potential predictors into a logistic regression, three variables left in the predictive model: the history of hypertension, AF rhythm, and dichotomous serum glucose level (cutoff value, 7.55 mmol/L, Table 3). The model depicted acceptable calibration (Hosmer–Lemeshow test, *P* = 0.554) and good discrimination (AUC, 0.853; 95% confidence interval, 0.775–0.930). The β-coefficients of the three predictors are listed in Table 3, and the illustration of the ISAT scale is shown in Figure 2. The optimal cutoff value of the predictive scale was 1 point with 95.1% sensitivity, 64.8% specificity, and 77.9% accuracy. The distributions and percentages of ISAT patients according to the predictive scale risk categories are illustrated in Figure 3. The percentage of ISAT increased with the rising ISAT score. ROC curves of the consecutive and dichotomous predictive scale are depicted in Figure 4.

**Table 3:** Predictors of *in situ* atherosclerotic thrombosis in the final multivariable regression model.

**Variable**	**ß-coefficient**	**Standard error**	**Wald**	**OR (95% CI)**	***P-value***
History of hypertension	1.649	0.611	7.283	5.203 (1.571–17.233)	0.007
Serum glucose level ≥7.55 mmol/L	1.564	0.566	7.646	4.778 (1.577–14.480)	0.006
Atrial fibrillation rhythm	−3.930	1.119	12.326	0.020 (0.002–0.176)	<0.001

*OR, odds ratio; CI, confidence interval*.

### External Validation

In the validation cohort (Table 4), the performance of the ISAT score was still promising, with an AUC value of 0.800 (0.682–0.918). When using 1 point as the optimal cutoff value, the diagnostic efficacy of the ISAT scale was 100% sensitivity, 54.3% specificity, and 68.6% accuracy. Performances of the ISAT scale in the validation cohort are depicted in Figures 3, 4.

**Table 4:** The characteristics of the validation cohort.

**Variable**	**Overall** **(*n* = 51)**	**The embolism group** **(*n* = 35)**	**The ISAT group** **(*n* = 16)**	***P-value***
Age, year, mean (SD)	63.6 (13.3)	65.0 (14.2)	60.6 (10.9)	0.281
Male, *n* (%)	36 (70.6)	21 (60.0)	15 (93.8)	0.014
Baseline SBP, mmHg, median [IQR]	156.0 [135.5, 170.0]	154.0 [135.0, 164.5]	164.5 [150.8, 179.0]	0.056
Baseline DBP, mmHg, median [IQR]	84.0 [76.5, 97.5]	83.0 [76.0, 90.0]	93.0 [81.5, 100.0]	0.066
Medical history				
Hypertension, *n* (%)	36 (70.6)	22 (62.9)	14 (87.5)	0.102
Diabetes mellitus, *n* (%)	12 (23.5)	8 (22.9)	4 (25.0)	1.000
Atrial fibrillation, *n* (%)	12 (23.5)	12 (34.3)	0 (0.0)	0.010
CHD, *n* (%)	7 (13.7)	5 (14.3)	2 (12.5)	1.000
Hyperlipidemia, *n* (%)	1 (2.0)	1 (2.9)	0 (0.0)	1.000
Smoking, *n* (%)	16 (31.4)	7 (20.0)	9 (56.2)	0.010
Baseline NIHSS score, median [IQR]	25.0 [17.0, 32.0]	25.0 [12.5, 32.0]	28.0 [20.0, 32.8]	0.304
Atrial fibrillation rhythm *n* (%)	15 (29.4)	15 (42.9)	0 (0.0)	0.002
Serum glucose level, mmol/L, median [IQR]	7.13 [5.58, 8.90]	7.11 [5.36, 7.80]	8.23 [6.53, 11.06]	0.025
Serum glucose level ≥7.55 mmol/L, *n* (%)	21 (41.2)	12 (34.3)	9 (56.2)	0.139
Intravenous thrombolysis, *n* (%)	2 (3.9)	2 (5.7)	0 (0.0)	1.000
OTP, min, mean (SD)	345.6 (211.1)	274.7 (127.1)	500.6 (273.1)	0.005

*ISAT, in situ atherosclerotic thrombolysis; SD, standard deviation; SBP, systolic blood pressure; IQR, interquartile range; DBP, diastolic blood pressure; CHD, coronary heart disease; NIHSS, National Institute of Health Stroke Scale; OTP, onset to puncture*.

## Discussions

The ISAT predictive scale consisted of dichotomous baseline serum glucose level, history of hypertension, and AF rhythm. This scale was convenient to use and had a promising predictive value for ISAT before EVT in acute VBAO patients.

As one of the most devastating subtypes of large vessel occlusion, the mortality rate of VBAO could be as high as 90% (23). No high-quality evidence favoring EVT has been established in VBAO (2, 24). Nevertheless, the superiority of EVT toward the best medical treatment is accumulating in studies worldwide (4, 5, 25), which shed light on this research area of uncertainty. ISAT is associated with a low recanalization rate and a high ratio of rescue therapy when compared with embolism-related occlusion (6, 26). The relationship between ISAT and poor prognosis is still debated (6, 8, 27, 28). Moreover, opinions on best treatment devices in ISAT populations are still controversial. Mechanical thrombectomy alone might not be sufficient enough to deal with ISAT and is associated with re-occlusion after EVT in ISAT patients (29). Some researchers emphasize the importance of angioplasty and stenting in treating intracranial atherosclerosis-related occlusion (30, 31). Disparities between ISAT and embolism-caused large vessel occlusion (LVO) urge neuro-interventionalists to judge the exact type of occlusion and choose the optimal treatment device. Furthermore, the basilar artery is one of the most common sites of atherosclerotic lesions (32). How to predict this subtype of VBAO before EVT is a crucial clinical question waiting for clinical researchers to answer.

So far, various hypotheses on discriminations of ISAT have been put forward. Truncal type occlusion (10), tapered occlusion (33), and occluded segment (34) were all proved to be indicators of intracranial atherosclerosis-related occlusion. Baseline DSA appearance was useful to distinguish underlying etiology, but the significance of predicting ISAT by using pre-EVT variables is much higher. Early prediction could help neuro-interventionalists and nurses get ready for following EVT, choose the best treatment devices, prepare additional therapies (e.g., glycoprotein IIb/IIIa inhibitor) in advance, and face the potential challenge with mental preparations.

In previous studies, ISAT patients are much prevalent in males, hypercholesterolemia, and posterior circulation involvement (9). The percentage of AF is significantly lower in the ISAT population (8). In our research, elevated glucose level, history of hypertension, and AF rhythm were independent indicators of ISAT. History of hypertension was prevalent in the ISAT group in our study, which was correlated with previous discovery: hypertension is a kind of risk factor of intracranial artery atherosclerosis (32). AF is a well-known risk factor for ischemic stroke and systemic embolism (35). In our model, active AF status was more efficient than the history of AF in distinguishing ISAT patients, which emphasized the importance of ECG examination on admission in identifying occlusion mechanisms. High glucose levels witnessed in the ISAT group correlated with previous research, as elevated blood glucose/hyperglycemia [fasting glucose ≥110 mg/dl (6.1 mmol/l)] is proved to be significantly associated with intracranial atherosclerosis (36), whereas the history of diabetes mellitus and glycosylated hemoglobin level were comparable between the ISAT group and the embolism group. This phenomenon might be attributed to the relatively small sample of this study. The deeper mechanism between ISAT and elevated glucose level still awaits further studies to answer.

Apart from variables left in the final model, other indicators might also be useful in distinguishing ISAT patients. ISAT is based on localized intracranial artery atherosclerosis. This long-lasting process might give additional time for collateral formation (28), and this might explain the reason why there is a lower percentage of severe stroke at the onset in the ISAT group.

Compared with previous studies, the most important strengths of our scale were convenient application, high sensitivity, and readily available parameters. It could be assessed within several minutes, and the accuracy was relatively high. The strengths of this study also included detailed data collection and credible assessments. External validation increased our persuasiveness.

However, it was worthwhile to mention the weaknesses of this study. First, potential recall and information bias were unavoidable in retrospective analyses, although various attempts (e.g., double-check the data collection process and independent evaluations) had been made. Second, a limited sample restricted the persuasiveness of our conclusion and the generalizability of the results. Third, relatively low specificity meant that some patients might be misclassified into the ISAT group and caused additional workloads for interventional doctors and nurses. Although the effectiveness of the ISAT scale was proved in the validation cohort with a lower ISAT percentage, it would be more practical to have the tools and medicines handy in the angio-suite regardless of the outcome of the scale, especially in a population where nearly half the cases were diagnosed as ISAT.

## Conclusion

The three-item predictive scale comprised of the history of hypertension, atrial fibrillation rhythm, and dichotomous baseline serum glucose level had a promising predictive value for ISAT before EVT in acute VBAO patients.

## Data Availability Statement

The raw data supporting the conclusions of this article will be made available by the authors, without undue reservation.

## Ethics Statement

The studies involving human participants were reviewed and approved by The Ethical committee of Jinling Hospital. Written informed consent for participation was not required for this study in accordance with the national legislation and the institutional requirements.

## Author Contributions

MZ, MW, RL, and XL designed the study. MZ, MW, XH, YJ, KH, and QY collected the data. MZ, MW, XH, YJ, XZ, RL, and XL analyzed and interpreted the data. MZ, MW, XH, XZ, KH, QY, HC, YJ, QL, DY, QD, RL, and XL drafted and modified the manuscript. All authors contributed to the article and approved the submitted version.

## Conflict of Interest

The authors declare that the research was conducted in the absence of any commercial or financial relationships that could be construed as a potential conflict of interest.
